# Iowa and Nebraska Veterinary Medical Association

**Published:** 1901-05

**Authors:** J. J. Drasky

**Affiliations:** Secretary


### SOCIETY PROCEEDINGS.

### IOWA AND NEBRASKA VETERINARY MEDICAL ASSOCIATION.

The second annual meeting took place at Omaha in one of the parlors of the Merchants' Hotel, at 2.30 P.M., on November 20, 1900. The meeting was called to order and presided over by President Dr. J. E. Brown, of Oskaloosa, Iowa. The Secretary and Treasurer, Dr. J. S. Anderson, of Seward, Neb., being absent, Dr. J. J. Drasky, of Crete, Neb., was duly elected Secretary and Treasurer *pro tem.* The minutes of the previous meeting were read, and after some corrections were approved. It was moved, seconded, and carried that the minutes should read that the committee on organization recommend that the temporary organization be made permanent, and that the members of the Iowa and the Nebraska Association in good standing were eligible to membership in this organization on paying a membership fee of fifty cents.

The President appointed the following Committee on Resolutions: Dr. A. T. Peters, of Lincoln, Neb.; Dr. H. E. Talbot, of Des Moines, Iowa, and Dr. M. V. Byers, of Osceola, Neb.

Under the head of new business, Dr. Peters made a most able speech in regard to the elevation of the army veterinarian. This question was discussed by other members present, and it was the opinion of them all that the Association should use its influence, and that the individuals should use their influence upon our Congressmen and Senators in behalf of the army veterinarian. It was moved, seconded, and carried that the Committee on Resolutions be instructed to draw up a set of resolutions to be sent to the Representatives in Congress asking them to use their votes and influence for the elevation of the army veterinarian.

Dr. Ramacciotti, of Omaha, Neb., president of the clinics, announced that the subjects would be ready on the afternoon of the following day, and that the clinics would take place in his infirmary.

It was moved, seconded, and carried that Dr. Ramacciotti be empowered to choose any of the members of the Association to assist him in the clinics. He chose Dr. Talbot, Dr. Drasky, Dr. Peters, and Dr. Gibson to operate on such subjects as he might assign to them.

The annual address was delivered by President Dr. Brown in an able and pleasing manner, and was commented upon by Dr. Talbot, Dr. Miller, Dr. White, Dr. Parstow, Dr. Drasky, Dr. Leslie, Dr. Vincent, and Dr. Everat.

Dr. J. G. Parstow, of Shenandoah, Iowa, read two papers—one on "Hyposulphite of Soda Poisoning," which elicited from quite a number of the doctors present a lively discussion, and another entitled "Cæsarean Section in the Cow."

A paper was read by Dr. J. H. Gain, of Seward, Neb., in which the doctor discussed an operation in the treatment of impervius urachus. While this is a new departure in the treatment of this trouble, it seems that the doctor has certainly found a radical cure which promises to do credit not only to its author, but to the entire profession as well. Many of the doctors present took part in the discussion of this interesting paper.

On motion, the Chair appointed Dr. Parstow, Dr. Gain, and Dr. Leslie an Auditing Committee, which was requested to report at once. The committee reported that the Secretary and Treasurer's books were in harmony with his report, and recommended the same for acceptance, which was at once voted upon and carried.

On motion the meeting adjourned to meet at 7 o'clock P.M. of the same day.

At 7.45 P.M. the Association was called to order by the President, and the following gentlemen were found present: Drs. J. E. Brown, J. J. Drasky, J. G. Parstow, H. E. Talbot, D. H. Miller, S. T. Miller, W. H. Austin, V. Shafer, M. V. Byers, W. A. Hammond, James Vinsent, Fred Evans, J. H. Gain, T. White, Bostrom, Gibson, Young, McKim, Crosford, Everat, H. Dell, and W. Thomas. Messrs. Duff, of Chicago; McIntosh, of the *Nebraska Farmer;* Janak, editor of *Hospadr;* Adams, John M. Pattar, J. W. Haxby, and S. L. Kostoryz, editor of *Osveta*.

Dr. Peters spoke on the subject of "Immunization of Hogs Against Hog-cholera." This speech was discussed by Drs. Vinsent, Gibson, Young, Drasky, Brown, Thomas, Miller, and Evans. It was moved, seconded, and carried that the Chair appoint a committee of six, with Dr. Brown as the chairman, to investigate and report on the subject of hog vaccination.

Dr. Ramacciotti reported that the last year's clinics had been a complete success, which was pleasing to the operators.

A somewhat heated discussion arose among the doctors on the subject, "What I Saw in Omaha." While this discussion took on an unpleasant aspect at times, yet it closed leaving the parties concerned on general good terms, and Dr. Drasky's action was approved by all the doctors present. It was hoped that no unpleasant or unfriendly relations existed after the discussion closed.

The report of cases was taken up by Dr. Thomas. In his report on parturient apoplexy the doctor confirmed his opinion expressed in a paper on the same subject read before the Nebraska Veterinary Medical Association some time ago, wherein he was of the opinion that we will find brain lesions on post-mortem examinations of. subjects dying of this disease. The doctor showed a brain with a large blood clot in the medulla oblongata. He urged that practitioners make post-mortem examinations of all subjects dying of this malady and keep a record of the same.

On motion, the meeting adjourned to give room to the Nebraska Veterinary Medical Association to meet again at 9.30 A.M. the following day.

SECOND DAY.—*November*, 21, 1900.

At 9.30 A.M. the President called the Association to order.

Dr. Drasky reported on a case of "Sorghum Poisoning, or What?" Discussed by Drs. Parstow, Miller, Peters, Lesley, Vinsent, Austin, Evans, and Brown. Dr. Gibson reported on "Hybrid-pumpkin Poisoning." Discussed by Drs. Lesley, Brown, Drasky, and Bostrom. Dr. Gain reported on a case of "Ridgling" in which the gland was found attached to the spleen. Dr. Gain also reported a case of "Melanosis."

Mr. Adams was introduced to the Association by Dr. Ramacciotti. Mr.

Adams is an old-time friend of the profession, and is the father of one of the veterinary bills which was passed in this State some years ago. He made an extensive and able address to the Association, in which he urged that the doctors continue in their struggle to secure legislation to the effect that the veterinary surgeon be recognized and protected by law. He very kindly offered his aid and his assistance to the Association and to the profession in their efforts along this line. Upon motion, Mr. Adams was made an honorary member of the Association by a unanimous vote, and a vote of thanks was also given him for his kindly offer of assistance to the Association.

The election of officers resulted as follows: President, Dr. Ramacciotti, of Omaha, Neb.; Vice-President, Dr. Austin, of Newton, Iowa; Secretary and Treasurer, Dr. J. J. Drasky, of Crete, Neb. The following Board of Censors were appointed by the Chair: Drs. Byers, Evans, Talbot, and Miller.

It was moved, seconded, and carried that the Secretary and Treasurer be instructed to pay Dr. Anderson $1.05 due him. It was moved, seconded, and carried that Dr. Ramacciotti be reimbursed for his expense of the clinics.

Dr. Parstow was appointed a committee of one to wait upon Dr. Ramacciotti and conduct him to the chair, where he was presented with the gavel by the retiring President. The new President delivered a short speech of acceptance.

Dr. Brown, the retiring President, delivered a very enthusiastic speech, which was greatly enjoyed by the Association.

A bill of $2 was allowed in favor of Dr. Brown, and the same was paid.

The Secretary was instructed to draw a set of resolutions thanking the Merchants' Hotel for the accommodations extended to the Association during their meeting, which was at once done.

The Association, on motion, adjourned to meet at 2 o'clock P.M. of the same day at the office of Dr. Ramacciotti.

At 2 o'clock P.M. the Association met in the office of Dr. Ramacciotti, where the clinics took place.

Dr. Ramacciotti was appointed to act as president of the clinics for the coming year, and he promised to have a great variety of subjects and everything in readiness so as to warrant the greatest thing in the shape of clinics yet undertaken in Omaha.

The Association, on motion, adjourned to meet next year at the call of the President and Secretary.

J. J. DRASKY,
Secretary.

